# Common yew intoxication: a case report

**DOI:** 10.1186/1752-1947-8-4

**Published:** 2014-01-02

**Authors:** Martin Vališ, Jaromír Kočí, David Tuček, Tomas Lutonský, Jana Kopová, Petr Bartoń, Oldřich Vyšata, Dagmar Krajíčková, Jan Korábečný, Jiří Masopust, Ludovít Klzo

**Affiliations:** 1 Department of Neurology, University Hospital in Hradec Králové, Sokolská 581, 50005 Hradec Králové, Czech Republic; 2 Charles University in Prague, Prague, Czech Republic; 3 Department of Emergency Medicine, University Hospital in Hradec Králové, Sokolská 581, 50005 Hradec Králové, Czech Republic; 4 Center forBiomedical Research, University Hospital in Hradec Králové, Sokolská 581, 50005 Hradec Králové, Czech Republic; 5 Department of Psychiatry, University Hospital in Hradec Králové, Sokolská 581, 50005 Hradec Králové, Czech Republic; 6 Department of Radiology, University Hospital in Hradec Králové, Sokolská 581, 50005 Hradec Králové, Czech Republic

**Keywords:** Yew intoxication, Extended cardiopulmonary resuscitation, Taxine alkaloid, Fatal poisoning

## Abstract

**Introduction:**

Taxine alkaloids cause fatal poisoning, in particular due to the compound’s toxic effect on the cardiovascular apparatus.

**Case presentation:**

We describe the case of a 39-year-old Caucasian man with common yew intoxication for whom cardiopulmonary resuscitation using all available methods, although delayed and extended, was successful.

**Conclusions:**

Extended and delayed cardiopulmonary resuscitation can be used successfully to treat common yew intoxication.

## Introduction

The toxicity of the European yew (*Taxus baccata,* family *Taxaceae*), a shrub that grows plentifully in the territory of the Czech Republic, has been known since ancient times and was known to the ancient Celts. The European yew contains several different compounds, such as phenolic compounds (for example, 3,5-dimethoxyphenol) together with non-alkaloidal diterpenoids (for example, 10-deacetylbaccatin III), alkaloidal diterpenoids (for example, paclitaxel, taxine B), flavonoids (for example, myricetin) and bioflavonoids (for example, bilobetin); some of these are considered as relatively highly toxic [1]. Descriptions of poisoning in the literature are quite rare. The rapid, lethal process of poisoning does not usually allow enough time for studying biochemical and electrophysiological changes. The plant’s poisonous parts include needles, bark and wood, but not the fruit (red berries). The taxine alkaloids (for example, taxine A, 2-deacetyltaxine A, isotaxine B, 1-deoxytaxine B) derived from *p*-dimethylaminohydroxycinnamic acid are the effective poisons of the yew [1]. In chemical terms, the compound is structurally related to veratrine, and the presence of an unsaturated lactone group makes this group of alkaloids similar to digitalis. Poisoning with the latter may be falsely diagnosed during a toxicological examination. Cardiac disturbances after intoxication by yew are ascribed mainly to the alkaloids paclitaxel and taxine B, affecting sodium/calcium permeability in cells [2]. The taxine alkaloid is absorbed through the digestive tract very rapidly, and the signs of poisoning manifest themselves after 30 to 90 minutes. An infusion made from 50 to 100g of needles is considered to be fatal [3-5], as no antidote is known.

## Case presentation

**Figure 1:**
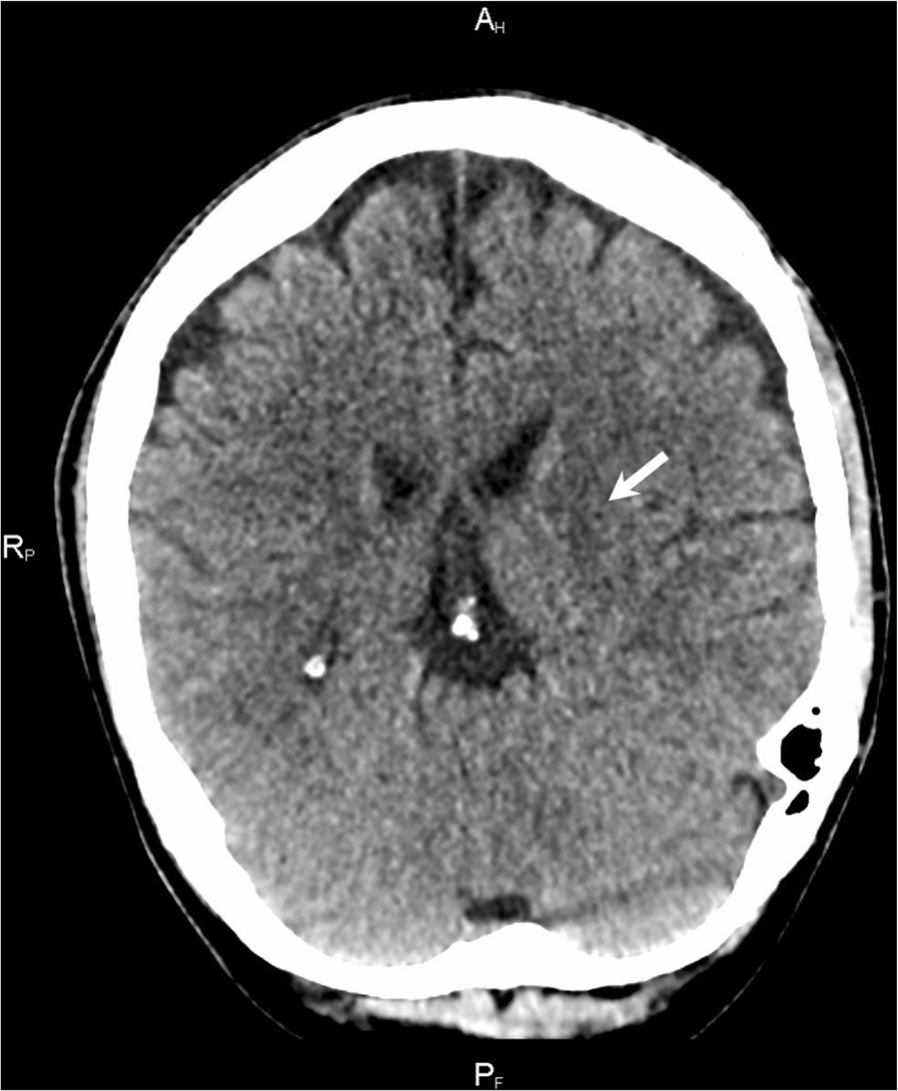
**Native computed tomography scan of the brain.** Slight hypodensity in the basal ganglia mainly on the left side (arrow) can be seen (post-ischemic changes after prolonged hypoxemia).

Having made a bet with his friend on whether yew or juniper is more poisonous, a 39-year-old Caucasian healthy man consumed a decoction from needles of the common yew (*Taxus baccata*) at approximately midnight. Gradually, weakness, nausea and vomiting began. Subsequently, clonic spasms developed in his extremities, followed by heart failure and blood circulation arrest. Urgent resuscitation, assisted via telephone, was started according to the instructions given by the dispatch center and lasted for five minutes. The emergency call was received at 7.30 a.m., and an ambulance arrived at 7.35 a.m. He arrived in the emergency department at 8.15 a.m., where bradyarrhythmia of 25 to 30 beats/min shifted to ventricular tachycardia (fibrillation) with repeated defibrillation. Indirect heart massage by means of an AutoPulse® (Zoll Medical Corporation, Chelmsford, MA, USA) device and extended cardiopulmonary resuscitation (CPR) were performed the entire time. Blood circulation was supported with noradrenaline 0.1mg/min intravenously. Adrenaline was repeatedly administered at a total dose of 6mg intravenously, plus sodium bicarbonate 300ml 8.4%, calcium gluconicum 10ml, calcium resonium 6 in graduated measures into a nasogastric tube, amiodarone 450mg intravenously and potassium and magnesium hydrogen aspartate 20ml intravenously. External and internal stimulation of the heart was ineffective. Taking into account the known cause of the blood circulation arrest and the probable absence of a delay in CPR, the introduction of venoarterial extracorporeal membrane oxygenation (VA-ECMO) was indicated as a rescue support therapy until the blood circulation was stabilized. DigiFab® (digoxin-reactive Fab protein, Protherics Inc., Brentwood, TN, USA) 120mg intravenously as an antidote was administered. Gradually, his blood circulation stabilized, and his heart rate was restored. Concurrently, clinical and laboratory signs of adult respiratory distress syndrome were observed. Corticotherapy with methylprednisolone 125mg intravenously once daily was started. VA-ECMO was successfully disconnected the next day; his oxygenation improved, and his Glasgow Coma Scale score was 3. An attempt to discontinue the use of sedation led to the development of generalized spastic activity. Our patient was evaluated for serious post-hypoxic encephalopathy with generalized myoclonic seizures. After a protracted, intensive rehabilitation period and symptomatic therapy, our patient was bedridden and breathing spontaneously, with minimal cortical reactivity and a persisting serious neurological deficit. It is not clear if our patient’s improvement was due to the therapy with DigiFab® or the result of supportive therapy. A native computed tomography (CT) scan of the brain showed slight hypodensity in the basal ganglia and post-ischemic changes after prolonged hypoxemia (Figure 1).

## Discussion

The success of treatment for poisoning depends on rapid reaction within the first half hour, when it is important to evacuate the stomach, through which the poison is absorbed, as rapidly as possible. This is because resorption from the acidic environment of the stomach is very rapid. The majority of afflicted individuals die within two hours of consuming yew, and serious symptomatology can develop as rapidly as within 30 minutes. The time available for rescue is thus very short in most affected individuals. First, it is necessary to evacuate the stomach as rapidly as possible by inducing vomiting or by means of stomach washout. From the very beginning, the development of ventricular tachycardia and ventricular fibrillation must be expected, which then shifts to terminal bradyarrhythmia and cardiac arrest in diastole. High-voltage discharge and temporary cardiostimulation may bridge the critical phase of poisoning. The possible effect of specific antidotes against digoxin is surprising, and can be logically explained by the chemical similarity of at least part of the complex of poisonous substances in the yew. Poisoning manifests itself as vertigo, nausea, abdominal pains, salivation, vomiting and diarrhea. Mydriasis is noticeable. The onset of somnolence, followed by unconsciousness, is frequent. The influence of taxine on the cardiovascular system is significant. Arterial hypotension commences as soon as the beginning of intoxication. Electrocardiography first usually shows supraventricular tachycardia, followed by ventricular tachycardia, with an early shift to ventricular fibrillation. The terminal rhythm is bradycardia, with an extreme expansion of ventricular QRS complexes. Intoxication terminates with cardiac arrest in diastole and, together with respiratory paralysis, is the immediate mechanism of death [3,4,6,7].

## Conclusions

Taxine alkaloids cause fatal poisoning, in particular due to the compound’s toxic effect on the cardiovascular apparatus. The present report describes a case of a man for whom CPR using all available methods, although delayed and protracted, was successful in treating yew poisoning.

## Consent

Written informed consent was obtained from the patient’s legal guardian for publication of this case report and any accompanying images. A copy of the written consent is available for review by the Editor-in-Chief of this journal.

## Abbreviations

CPR: Cardiopulmonary resuscitation; VA-ECMO: Venoarterial extracorporeal membrane oxygenation.

## Competing interests

The authors declare that they have no competing interests.

## Authors’ contributions

MV was a major contributor in writing the manuscript. JKoč, DT, TL, JKop and PB performed cardiopulmonary resuscitation. OV and DK performed neurological treatment. JK and JM performed specialized care. LK analyzed and interpreted the computed tomography findings. All authors read and approved the final manuscript.

## References

[B1] Wilson CR Sauer J Hooser SB Taxines: a review of the mechanism and toxicity of yew (*Taxus* spp.) alkaloids Toxicon 2001 39 175 185 10.1016/S0041-0101(00)00146-X 10978734

[B2] Thuret-Carnahan J Bossu JL Feltz A Langley K Aunis D Effect of taxol on secretory cells: functional, morphological, and electrophysiological correlates J Cell Biol 1985 100 1863 1874 10.1083/jcb.100.6.1863 2581977 PMC2113595

[B3] Wehner F Gawatz O Suizidale Eibenintoxikationem - von Caesar bis heute - oder Suizidanleitung im Internet [in German] Arch Kriminal 2003 211 19 26 12635487

[B4] Willaert W Claessens P Vankelecom B Vanderheyden M Intoxication with Taxus baccata: cardiac arrhythmias following yew leaves ingestion PACE 2002 25 511 512 10.1046/j.1460-9592.2002.00511.x 11991380

[B5] Smit MR Taxus baccata poisoning in lambs and meat inspection Tijdschr Diergeneeskd 1992 117 697 699 1462346

[B6] Tekol Y Kameyama M Elektrophysiologische Untersuchungen uber den Wirkungsmechanismus Eibentoxins Taxin auf das Herz [in German] Arzneimittelforschung 1987 37 428 431 2440454

[B7] Feldman R Chrobak J Liberek Z Stajewski J Four cases of poisoning with the extract of yew (Taxus baccata) needles Pol Arch Med Wewn 1988 79 26 29 3275166

